# Evaluation of the NFHS Online Captains Leadership Course: Student Athletes’ Views of Effectiveness

**DOI:** 10.3389/fpsyg.2021.648559

**Published:** 2021-03-24

**Authors:** Lauren F. Walker, Daniel R. Gould

**Affiliations:** ^1^Department of Exercise Science, Elon University, Elon, NC, United States; ^2^Department of Kinesiology, Institute for the Study of Youth Sports, Michigan State University, Lansing, MI, United States

**Keywords:** leadership, youth, sport, captain, life skills

## Abstract

Sport is viewed as an arena for positive life skill development, including leadership development. In 2015, the NFHS launched an online Captain’s Leadership Training Course. The main purpose of this study was to examine the effectiveness of the course in improving leadership knowledge and ability. An electronic survey was sent to a sample of athletes (*n* = 202, 129 female), ages 13–19 (*M* = 17.01, SD = 0.10) in eight United States states who had completed the NFHS course within the last 3–18 months. Most athletes (92.6%) completed the course based upon their coach’s recommendation. The course was viewed to be moderately to very useful (*M* = 2.49, SD = 1.00) in helping them in preparing to be a team captain. Participants believed the course to be very to extremely effective in building their knowledge on motivation (*M* = 1.96, SD = 0.89), communication (*M* = 1.90, SD = 0.80), decision making (*M* = 2.03, SD = 0.91), peer modeling (*M* = 1.91, SD = 0.86), team cohesion (*M* = 1.96, SD = 0.88) and problem solving strategies (*M* = 2.00, SD = 0.85). Canonical correlation analyses showed that athletes who felt they were more reflective tended to rate the effectiveness of the course lower than their peers. Additionally, analyses did not show any clear demographic characteristics that distinguished between perceptions of the effectiveness of the course, showing the value found in the course was high with all types of scholastic athletes. Athletes felt the course could be improved most in the area of learning how to manage conflict with their peers and coaches. Future research in scholastic leadership should seek to understand the impact of the course prospectively across a high school sport season.

## Introduction

Sport has long been viewed as an avenue for facilitating positive youth development *via* the promotion of life skills (Gould and Carson, 2008; Camiré et al., 2011; Watson et al., 2011). Among these potentially transferable skills, which include such things as resilience, goal setting, character development and integrity, is leadership (Gould et al., 2006). The extensive body of research in the arena of leadership within the business, education and the sport context highlight the perceived importance of this skill in our culture (Voelker et al., 2011; Gould et al., 2012). Discussions of leadership, as a life skill, are also unavoidable within the formal contexts of athletic teams, due to the presence and tradition of captaincy (Gould et al., 2012). In addition, coaches continually refer to quality leadership as one of the most critical elements in effective team performance, team motivation, and team unity, citing leaders as an “extension” of the coaching staff (Bucci et al., 2012; Gould et al., 2013). In fact, coaches appear to value quality leadership to such an extent that they cited poor athlete leadership as one of the top six biggest issues with adolescent athletes (Gould et al., 2012). However, Gould et al. (2006) emphasized that the development of positive life skills does not occur simply through involvement in sport; it must be an intentional focus of the sport program to produce the desired outcomes.

While there is an abundance of leadership research focused on the characteristics and impact of effective coach leadership (Côté and Gilbert, 2009; Vella et al., 2010), there is still much that is unknown about cultivating quality athlete leadership. Youth leadership research often falls into examinations of formal athlete captaincy (e.g., Gould and Voelker, 2010, 2012; Voelker et al., 2011; Gould et al., 2013) or examinations of informal leadership roles and the development of leadership skills outside of the captaincy designation (e.g., Fransen et al., 2014, 2015, 2019). Regardless of the view taken, even a formal captaincy designation does not guarantee intentional leadership development in these youth athletes, with Gould and Voelker (2010, 2012) reflecting that leadership development is highly variable across athletic teams, ages, and skill levels. In addition to the transfer of these skills outside of sport, research reflects quality athlete leadership is connected directly to performance outcomes and indirectly to team cohesion, satisfaction in sport, and collective efficacy which can be mediators of performance outcomes (Carron et al., 2002; Chow and Feltz, 2007; Price and Weiss, 2013; Bruner et al., 2014; Filho et al., 2014; Cotterill and Fransen, 2016).

Due to the potential impact effective athlete leadership can have on team outcomes, it is important to understand how to intentionally develop leadership skills (Cotterill and Fransen, 2016). Several recent studies have tested interventions designed to enhance athlete leadership. Using a case study methodology, Cotterill (2017) implemented a leadership development program for elite cricketers with the intervention targeting captaincy development, leadership skills, and personal growth. Evaluation data was assessed *via* player feedback, staff feedback, and the consultant’s own reflections on the program. The findings suggested that the program was impactful and beneficial in fostering leadership in the players. However, due to both the unique culture of cricket (e.g., the autonomy and importance of the athlete leader) and the elite level of competition, Cotterill’s (2017) program design may not offer a complete transfer to a youth athlete population.

In another study, Duguay et al. (2016) evaluated a season-long leadership development program using 27 female varsity collegiate athletes. All the athletes took part in a series of four workshops focused on such behaviors as being an appropriate role model, using demographic behaviors, providing positive feedback, and elements of transformational leadership such as inspirational motivation and individual consideration. Pre- versus post-intervention assessments revealed that significant differences emerged in leadership behaviors, peer motivational climate, and athlete satisfaction due to the intervention. Similarly, Voight (2012) implemented a collegiate leadership development program using one male and one female athletic team. Voight’s (2012) intervention focused on improving team communication, *via* teammates deciding on objectives for the team collectively and expressing their needs/feedback to leadership, personal leadership reflection, and weekly meetings educating team leaders on the ways to accomplish responsibilities and solve problems. Voight’s (2012) assessment of the program found that athletes felt the time was well spent and that the intervention did influence team performance, cohesion, and personal leadership skills.

Similar to Cotterill’s (2017) case study, the findings from Voight’s (2012) and Duguay et al.’s (2016) studies may be limited in their transferability to another team culture or level of competition, like the youth athlete, due to the unique demands of the collegiate setting. However, one important commonality between the programs mentioned was the presence of a reflection component in each intervention program (Voight, 2012; Duguay et al., 2016; Cotterill, 2017), highlighting the potential importance of reflecting in action for athlete leaders. While more intervention studies are needed, these initial studies are encouraging in that they provided evidence that athletes can learn to lead *via* formal programming. Additionally, changes in leadership behavior cited were associated with key outcomes such as team cohesion, motivational climate, and communication (Voight, 2012; Duguay et al., 2016; Cotterill, 2017). However, as these interventions were run by researchers, external to the team, more needs to be known about how sport coaches can directly facilitate leadership development on their teams.

Preliminary knowledge of the role of the coach in athlete development came from the work of Wright and Côté (2003). Wright and Côté (2003) highlighted the need for coaches to be kind and supportive, develop physical skills and understanding of the game, provide opportunities to advance in sport, assign specific roles, and include the athletes in important leadership decisions, if they wanted to optimally develop athlete leaders. Indeed, this last point was emphasized in an intervention leadership program that was developed by Blanton et al. (2014). Within this intervention, the importance of empowering the athlete leader to make decisions, and take ownership over the results of those decisions, was key in developing effective leadership behaviors (Blanton et al., 2014). Furthermore, in studying scholastic coaches known for developing leadership in their captains, Gould et al. (2013) identified that using formal leadership courses, allowing leaders to make decisions regarding team goals, and consistently prioritizing communication between coach and leaders as important best practices. However, a follow-up study by Voelker et al. (2019) surveyed scholastic coaches across the United States and showed that while almost 90% of the coaches felt formal leadership development programs could be useful, only 12% cited using such programs to build their athletes’ leadership skills.

As such, while the above recommendations are valuable for coaches in intentionally training their leaders, there is still a disconnect between the number of coaches that purport to value leadership development and those that make it a priority of their program (Voelker et al., 2011, 2019; Gould et al., 2013). A major barrier to the implementation of leadership development on the part of coaches is a lack of time (Gould et al., 1999; McCallister et al., 2000; Voight, 2005; Paquette and Sullivan, 2012). For many, high school coaches in particular, coaching is not their full-time job. This factor, along with family life, presents a very real barrier on the amount of time with which coaches have to prepare for all of the responsibilities of their roles (Voight, 2005; Camiré et al., 2011). Not only is time a barrier but coaches may not have the knowledge or coaching efficacy to implement a leadership development program for their athletes (Voelker et al., 2019). In the end, whether the reason for not engaging in leadership development is due to a lack of knowledge or simply not having enough hours in the day to make it a priority, there needs to be a focus on promoting both the importance of leadership development to successful sporting outcomes and resources for developing leadership that are easy to access and use for the coach and athlete.

One leadership development resource that currently exists, which may circumvent the above-mentioned challenges, is the National Federation of State High School Association’s (NFHS) Online Captain’s Course. This course was developed in partnership with the Michigan High School Athletic Association (MHSAA) and the Institute for the Study of Youth Sport (ISYS) at Michigan State University and launched in March 2015. While the course was not based on one specific leadership theory, as many of these theories are targeted at the adult leader, it was informed by van Linden and Fertman’s (1998) notions of how youth learn to lead by first seeing themselves as a leader, developing leadership skills, practicing those skills, and finally reflecting on what was learned. Additionally, the course content was supplemented by youth sport research in the area of athlete leadership and loosely structured after the long-standing MHSAA-ISYS in-person captains’ clinics that have become a staple of the Michigan athletic landscape (Gould and Voelker, 2010; Pierce et al., 2018).

While not a prerequisite, the NFHS course was designed for those student athletes who are team captains or those with a formally recognized leadership role (Pierce et al., 2018). However, youth athletes do not need to be a captain, or a scholastic sport athlete, to take the NFHS course and grow as a leader. The goal of the course is to provide the student athlete with an opportunity to reflect on their current or potential role as a leader on the team, while providing tangible knowledge, skills, and strategies to use on the field. The course features first-person accounts of other student-athletes about their experiences as leaders, *via* on-screen hosts and captain interviews (Pierce et al., 2018). It consists of 10 “chapters,” is offered free to charge, and takes roughly 4 h for the athlete to complete (Pierce et al., 2018). While the course was designed to be online, in an effort to reach more student athletes in the absence of a coach, the designers felt that the course alone was not enough. Hence, a coach’s guide for supporting and contextualizing the course information to their sport environment is available at no charge (Pierce et al., 2018).

Since its launch in 2015, the online course has been completed roughly 38,000 times online, which appears to show some promise for easy access and utilization of the resource with formal and informal team leaders alike. This resource also shows promise as a low time requirement resource, from which coaches can start developing leadership programs for their athletes. While the online captain’s course is research based, it has not yet been evaluated for its effectiveness in helping youth athletes understand their roles as a team leader and the necessary leadership knowledge and strategies that may benefit them on the playing field. As such, two purposes guided this study: (1) to evaluate the course’s effectiveness in improving athlete knowledge about becoming a sport leader, and (2) to determine if athlete’s responses were influenced by demographic characteristics and their reflective ability.

## Materials and Methods

### Participants and Participant Selection

To address the study purposes an electronic survey design was used, incorporating both closed and open-ended questions. Data were collected with scholastic student athletes who completed the NFHS Online Captain’s Course within 18 months of the data collection. Prior to contacting participants, the study was approved by the Institutional Review Board, the NFHS, and the state athletic associations for the eight states used in the study. The eight states chosen, California, Florida, North Carolina, Maryland, Massachusetts, Michigan, Ohio, and Rhode Island represented the states with the most athlete completions of the Captain’s Leadership Course. After obtaining the necessary approvals, participants were contacted *via* the e-mail they provided when signing up for the course. Participants were sent three reminders, 1 week apart, to participate in the survey. Participants under the age of 18 had to electronically obtain parental consent and provide assent to participate in the study before they could access the survey. Participants over the age of 18 were asked to provide electronic consent before they could access the survey. Participants could cease participation in the study at any time and were provided a $10 Amazon gift card for their participation.

After providing consent, participants were provided with a summary page, reminding them of the basic elements of the NFHS Captain’s Course content. To view the summary of the topical content from the course, see Table 1 below. The only necessary inclusion criterion was that athletes had to have completed the NFHS Captain’s Course in its entirety. Two hundred forty-nine athletes agreed to participate in the study; however, only 202 completed the survey in its entirety, reflecting an 81.5% completion rate.

**TABLE 1:** Summary of NFHS course modules.

Module 1: Introduction to leadership (5 sections)
Module 2: Who am I as a student-athlete? (6 sections)
Module 3: What is my leadership style? (5 sections)
Module 4: What are my roles and responsibilities? (4 sections)
Module 5: Positive peer modeling (6 sections)
Module 6: Communication (4 sections)
Module 7: Motivation (5 sections)
Module 8: Team building and team cohesion (6 sections)
Module 9: Handling tough situations (3 sections)
Module 10: Leadership in review (1 section)

*Each section is approximately 10 min in length and takes roughly 4 h to complete.*

### Instrumentation

This survey incorporated: (1) demographics variables; (2) evaluations of the course; and (3) Kember et al.’s (2000) Reflection and Critical Reflection subscales. In addition, to the quantitative scales and questions incorporated with the survey, select open-ended questions were asked of the athletes (e.g., please give an example of something you were able to use from the course). All verbiage on the survey was altered to no greater than a 6th grade reading level, to fit the youth audience, with the exception of the items in Kember et al.’s Reflective Subscales.

#### Demographic Variables

The following demographic variables were obtained from the sample of captains that completed the course: age, last year completed in school (as some of those surveyed completed the survey during summer/after they graduated from high school), gender, the sports in which they participated, time since completion of the course (verified by the completion date provided with the e-mails from NFHS), whether or not their coach knew they took the course, and the extent to which coaches were involved in the course.

#### Leadership Variables

The leadership variables gathered on the survey were meant to provide context to the previous leadership experiences that occurred before taking the NFHS course that may have influenced each sport leader. The following leadership variables were gathered with the survey: previous experience serving as a sport leader, experience as a leader in other organizations outside of sport both in (e.g., student government) and outside of school (e.g., Boy/Girl Scouts), and experience with other formalized leadership training prior to the course. Rather than treat these items separately, the four items were combined to calculate a “total leadership score” which represented a compilation of prior leadership development. To create this “leadership score” each listed leadership experience/training was given a designation of 1 and all cited experiences were then summed for the individual’s score. For example, if “Sarah” had a total leadership score of 3, this came from: being a captain of a club team (1), being a student government rep (1), and being a Girl Scout leader (1). Additionally, athletes were asked to rate the degree to which they viewed themselves as a leader and the age at which they first felt they could be a leader.

#### Kember et al. (2000) Reflective Subscales

A part of leadership that has often been cited as necessary for effectiveness is the ability to be reflective. As such, **Kember et al.’s (2000)** Reflection and Critical Reflection subscales were used to evaluate: (1) the level to which athletes who took the NFHS course felt they were reflective individuals; and (2) if the course itself made them more reflective about their leadership positions. All responses were provided on a Likert-type scale, with 5 being “strongly agree” and 1 being “strongly disagree.” As the reflective subscales were not designed to pertain to leadership specifically, the wording of items was adjusted slightly, and internal consistency was checked on the subscales to ensure reliability and validity of the measures for this sample.

#### Course Evaluation Questions

The development of the NFHS course was part of a larger leadership training initiative between the ISYS, at Michigan State University, and the MHSAA. The individuals that developed this course based its development upon six primary areas of leadership development deemed relevant to high school sports- motivation, communication, decision-making, peer modeling, team cohesion and solving problems (Pierce et al., 2018). Athletes were asked to respond to evaluative questions about the course, which include questions regarding the overall usefulness of the course in preparing them to be a leader, effectiveness of the course in helping them understand what leadership entails, and the course’s effectiveness in helping them develop the skills to be a leader both in and out of sport. They were then asked about the six sport-specific areas of leadership above and asked to rate how much the course helped them to improve their knowledge in these areas. All responses were provided on a 5-point Likert scale, with 1 being “extremely good” and 5 being “not at all good.”

#### Open-Ended Questions

Open-ended questions were included to gain a fuller understanding of opinions about the NFHS course. As such, the following questions were asked: (1) what characteristics make an effective leader, (2) how they found out about the course, (3) what motivated them to take the course, (4) an example of something they used from the course, and (5) any feedback they had regarding elements of the course that should be changed or added.

### Data Analysis

Due to the large number of variables collected in this survey, data analysis was driven primarily to address the two purposes of the study: (1) to evaluate the course’s effectiveness in improving athlete knowledge about becoming a sport leader; and (2) to determine if athlete’s responses were influenced by demographic characteristics and their reflective ability. To examine these purposes descriptive statistics were calculated and a canonical correlation was conducted, respectively. Factor analyses were performed, where necessary, to validate and ensure reliability for scales used in the study. Furthermore, thematic content analysis was used to create meaning units and themes in the open-ended response data.

## Results

### Demographics

Two hundred three total participants completed the electronic survey, 129 females and 74 males. The age of participants ranged from 13–19 years, with the mean age of 17.01 (SD = 1.0). The last grade completed followed suit with age, with the mean being between 11th and 12th grade (*M* = 11.61, SD = 0.68). This mean age was unsurprising given the typical age at which high school athletes are first given leadership positions on their teams- junior and senior years. A broad range of sports were represented with track and field (*n* = 77), soccer (*n* = 50), cross country (*n* = 49), and basketball (*n* = 41) most represented in the sample. However, it should be noted that several participants were multi-sport athletes and 18 total sports, individual and team-based, were represented in the sample.

In regard to their previous leadership experiences, the vast majority of athletes (89.6%) had served as a captain of a high school sport team before taking the survey, with the mean number of teams captained being 1.33 (SD = 0.81). The average number of non-sport high school leadership positions (e.g., student government, NHS) was slightly lower, 1.13, (SD = 1.42), with roughly half (49.3%) of participants not serving in any non-sport high school leadership positions. The same trend held for out of school leadership positions (e.g., Boy/Girl Scouts, jobs) with only 43% of athletes citing a leadership position (*M* = 0.80, SD = 1.12). Finally, when asked about any formal leadership training (e.g., leadership clinics or summits) they had received prior to the NFHS course, 32% of participants cited engaging in a leadership training course (*M* = 0.35, SD = 0.56).

In understanding the athletes’ evaluation of the course retrospectively, the amount of time between taking the course and completing the survey was obtained. Eighty-nine athletes in the sample had taken the course in the last 6 months, 49 within 6–12 months, and 65 completed the course more than 12 months ago. Furthermore, athletes were asked about the level of coach involvement they received while completing the course. Most athletes (93.1%) completed the course at the request of their coach. Within this 93%, 50 took the course because it was their coach who recommended it, with 10 of these coaches providing some sort of follow-up on the course content; 136 athletes took the course because their coach required it, with 52 of these coaches providing some sort of the follow-up on the course content. More specific motivations for completing the course can be found in Table 2.

**TABLE 2:** Motives for taking the NFHS leadership course.

**To complete a requirement (*N* = 105)**
Coach (*N* = 60)
Unspecified requirement (*N* = 29)
School/Athletic Director (*N* = 16)
**To learn more about leadership in sport (*N* = 74)**
To improve personal leadership skills (*N* = 22)
Wanted to be a captain this or next year (*N* = 20)
To become a better captain (*N* = 14)
Wanted to learn about leadership and thought it would be interesting (*N* = 11)
To better self as much as possible (*N* = 7)
**Miscellaneous reasons (*N* = 6)**
The desire to become a coach (*N* = 1)
Want to make a change in my team (*N* = 1)
The loss of influential leaders on the team (*N* = 1)
A passion for learning (*N* = 1)
It was a great opportunity (*N* = 1)
Looked good on my college application (*N* = 1)

### Reflective Ability Subscales

Both the Reflection and Critical Reflection subscales of Kember et al.’s Reflection Scale were included in the survey. Internal consistency was evaluated for both subscales *via* Cronbach alpha. Both subscales achieved acceptable reliability for α = 0.72 for the Reflection subscale and α = 0.85 for the Critical Reflection subscale.

The Reflection subscale asked participants to rate how reflective the were as an individual, with 5 being “definitely agree” and 1 being “definitely disagree” with total scale scores ranging from a low of 5 to a high of 20. On average, athletes rated themselves as reflective individuals (*M* = 17.54, SD = 2.17).

While the Reflection subscale focused on athletes’ perceptions of their own reflective ability, the Critical Reflection subscale asked participants to rate how much the course itself made them reflective. Athletes rated the course as making them somewhat reflective, (*M* = 14.66, SD = 3.97) as a score of 20 indicate the participant “definitely agreed” with every subscale statement. The modified items and individual item descriptive statistics can be seen in Table 3.

**TABLE 3:** Reflective subscale scores.

	*M*	SD
**Reflection subscale**		
I sometimes question the way other do something and try to think of a better way.	4.40	0.66
I like to think over what I have been doing and consider alternative ways of doing it.	4.33	0.79
I often reflect on my actions to see whether I could have improved on what I did.	4.56	0.61
I often re-appraise my experience so I can learn from it and improve for my next performance.	4.36	0.71
**Critical reflection subscale**		
As a result of this course I have changed the way I look at myself (as a leader).	3.99	1.07
This course has challenged some of my firmly held ideas (about leadership).	3.38	1.27
As a result of this course I have changed my normal way of doing things (and leading).	3.71	1.10
During this course I discovered faults in what I had previously believed to be right (about being a leader and leading).	3.67	1.20

*5 = definitely agree to 1 = definitely disagree.*

### Course Effectiveness Findings

Participants were asked to answer a total of 11 evaluation questions regarding different aspects of the course. Athletes rated the overall usefulness of the course between “moderately” and “very” useful (*M* = 2.49, SD = 1.00) on a 5-point Likert-type Scale, with 1 being “extremely useful” and 5 being “not at all useful.” The top-rated areas of the course were: motivation, communication, decision making, peer modeling, team cohesion and solving problems. These modules in the NFHS course were primarily centered in assisting athletes in improving their leadership in these highly sport relevant topics. Ratings on these items ranged from “very” to “extremely” useful (*M* = 1.90–2.03, SD = 0.80–0.91). Full course ratings can be found in Table 4.

**TABLE 4:** Course evaluation ratings for NFHS captain’s leadership course.

	*M*	SD
**Evaluation questions**		
How useful did you find the National Federation Captain’s Leadership Training course to be in preparing you for leadership roles?	2.49	1.00
How effective was the course in helping you understand what leadership is?	2.21	0.93
How effective was the course in helping you understand important components in effective leadership?	2.07	0.86
Overall, how effective was the course in helping you develop the skills to be a leader in sport?	2.10	0.98
Overall, how effective was the course in helping you develop the skills to be a leader outside of sport?	2.45	1.04
**How effective was the course in improving your knowledge as a captain/leader in each of the following areas:**		
Motivation	1.96	0.89
Communication	1.90	0.80
Decision making	2.03	0.91
Peer modeling	1.91	0.86
Team cohesion	1.96	0.88
Solving problems	2.00	0.85

### Canonical Correlation

A canonical correlation analysis was conducted to address the second purpose of this study, determining the relationship between personal and leadership related variables and evaluations of the course. Canonical correlation was chosen due to the analysis’ ability to highlight the strength of a relationship between sets of variables. For this analysis, gender, total leadership experience score (see calculation in section “Materials and Methods”), the length of time since completing the course, the athlete’s view of their ability to be a leader, and their Reflection subscale score served as predictor variables, and the overall usefulness of the course, the effectiveness in increasing knowledge on motivation, communication, decision making, peer modeling, team cohesion, and problem solving, and their score on the Critical Reflection subscale were used as criterion variables.

A significant canonical relationship between the two sets of variables did emerge from the analysis, Wilkes λ = 0.700, *F*(40, 818) = 1.741, *p* < 0.05. Only one significant canonical function emerged, R_c__1_ = 0.397, reflecting 15.8% overlapping variance. While a significant relationship was present between the two sets of variables, the function suggested a weak correlation. The redundancy index reflected that 38% of the variance in evaluations were explained by the predictor variables, which meets Tabachnick and Fidell’s (2007) recommendation of at least 10% to be deemed significant and meaningful. So, while the relationship was weak overall between predictor and criterion variables, it was still deemed significant and meaningful.

In examining the relative contribution of each predictor variable to the multivariate relationship (Table 5) per Tabachnick and Fidell’s (2007) recommendations of 0.30 cut points for meaningful contributions to the relationship, only the total leadership experience score (loading = −0.35) and self-rated perceptions of reflectivity score (loading = 0.82) meaningfully contributed to the set of evaluative scores regarding the course. Among the criterion variables, all evaluations of the course contributed meaningfully to the relationship, although improving knowledge of decision making (−0.82), motivation (0.70) and critical reflection (0.75) were the variables with the highest canonical weights. As such, this canonical analysis revealed that that those student athletes who perceived themselves to be more reflective individuals pre-course rated the course lower overall, but still felt it made them more reflective about leadership. In regard to total leadership score, the higher the leadership score rating (i.e., the more previous experiences an athlete had prior to the course), the higher their evaluation of the course. This result seems to suggest that even those with previous leadership training still found value in a course directed a sport-specific leadership.

**TABLE 5:** Factor loadings for relationship between demographic characteristics and course evaluations.

Factor	1
**Predictor variables**	
Gender	−0.15
Total leadership experience score	−0.35
Reflection scale ratings	0.82
Date course taken	−0.003
View of self as leader	−0.10
**Criterion variables**	
Overall usefulness of course in preparing to be team leader	−0.44
How effective course was in improving knowledge on motivation	−0.70
How effective course was in improving knowledge on communication	−0.44
How effective course was in improving knowledge on decision making	−0.82
How effective course was in improving knowledge on peer role modeling	−0.55
How effective course was in improving knowledge on team cohesion	−0.41
How effective course was in improving knowledge on problem solving	−0.68
Critical reflection subscale	0.75

### Open Ended Qualitative Responses-Specific Uses of the Course and Improvements

In addition to asking athletes for their quantitative ratings of the effectiveness of the course, several open-ended questions were posed to better understand what led them to and what they were able to use from the course. All responses to open-ended questions were compiled and grouped thematically into like responses. The number of athletes providing similar responses were also noted (See Tables 6, 7).

**TABLE 6:** Frequency of athlete examples of course content most able to use.

**Communication module (*N* = 43)**
How to better communicate/mentor new members of the team (*N* = 23)
How to be the link between coach and teammates (*N* = 11)
Better listening skills and relationships with teammates (*N* = 9)
Athlete quote: *“I never leave practice now without communicating to each of my teammates about how hard they worked and things that need to be improved.”*
**Motivation module (*N* = 37)**
Motivation and encouragement of teammates (*N* = 32)
How to set goals (*N* = 5)
Athlete quote: *“I was able to use the real-life stories of captains (from the course) as motivation for myself to model them. They gave me energy to want to be a good captain.”*
**Handling tough situations and decision-making module (*N* = 34)**
Handling problem teammates with confidence and calmness (*N* = 23)
How to make decisions as the leader (*N* = 11)
Athlete quote: *“There was an instance on my team where two girls got in a fight outside of practice, and I was able to bring it to the coach’s attention and talk to the girls to work out the situation.”*
**Cohesion module (*N* = 28)**
Learning how to build each of the specific types of team cohesion (*N* = 28)
Athlete quote: *“I feel that the content of the course helped me with team cohesion. We had some new players this year, and the material helped me in getting my teammates to work together and gel. As a result, our season was much more successful than expected.”*
**Modules regarding leadership definition and roles/responsibilities (*N* = 13)**
How to lead my peers with confidence and be a persuasive leader (*N* = 8)
Different types of leaders and the roles and responsibilities of captains (*N* = 5)
**Positive peer modeling module (*N* = 8)**
How to be a positive peer model for young athletes/kids (*N* = 8)
Athlete quote: *“Because I had taken the course, I knew that even though I was hurt I needed to go to every practice and every game to show my team that I supported them. If I could come, even though I was hurt, they could do the same.”*

**TABLE 7:** Recommendations for improving the course.

More realistic scenarios and problem-solving strategies, especially with resolving conflict (*N* = 17)
Ways to navigate conflict with the coach (*N* = 10)
More interactive format, rather than just videos and writing (*N* = 7)
More examples of how to build both types of team cohesion (*N* = 5)
More examples/leaders from outside of sport (*N* = 3)

An inspection of Table 6 reveals that the course modules most often used in real-time leadership situations were communication, motivation and handling tough situations. Regardless of the module, specific topics cited as most often used were: Motivation and encouragement of teammates (*n* = 32); learning how to build the specific types of team cohesion (*n* = 28); how to better communicate/mentor new members of the team (*n* = 23); and, handling problem teammates with confidence and calmness (*n* = 23).

Table 7 reflects participant’s responses when asked what could be changed or added to the course to make it more effective in enhancing their sport leadership skills. Athletes had a strong desire to see more realistic scenarios, especially relative to resolving conflict with their peers and coaches (*n* = 27). Furthermore, athletes desired for the course to be more interactive compared to the one-sides format of videos and writing reflections (*n* = 7). This desire for further real-time or dual interaction with more than the computer interface, in conjunction with the desire to learn conflict resolution skills, may indicate some of the limitations with an electronically based course offered to a wide audience.

## Discussion

This study aimed to examine the utility of a leadership development resource available to scholastic youth athletes- the NFHS Online Captains Course. To better understand the usefulness and effectiveness of this course in building athlete leadership, two purposes guided this study: (1) to evaluate the course’s effectiveness in improving athlete knowledge about becoming a sport leader, and (2) to determine if athlete’s responses were influenced by demographic characteristics and their reflective ability.

### Athlete Perception of the NFHS Course

In addressing the first purpose, study participants rated the course overall as “moderately” to “very useful” in preparing them for their leadership role, with the specific modules of communication, peer modeling, motivation and team cohesion being judged as the content areas that most improved the participant’s knowledge. Open-ended response data supplemented the quantitative findings; the same modules were mentioned as athletes elaborated specific strategies that they used in their leadership on the playing field. The overall positive perception of the course is a promising finding in light of the fact that the program is available nationally and has been completed by almost 38,000 high school athletes. It is reassuring to know that the users find the course to be useful in their sport leadership growth.

In examining the second purpose of the study, whether demographic and background factors influenced how effective student athletes found the course, canonical correlation analysis revealed that those student-athletes who perceived themselves as more reflective individuals rated the course lower, but still felt that the course made them reflective about their leadership. This finding is curious but may encompass the idea that an athlete’s perception of their own reflectivity may impact their openness to learning new material. Due to the frequency with which reflection is included in athlete leadership development courses (Voight, 2012; Duguay et al., 2016; Cotterill, 2017), and its potential importance in leadership growth, the role of this variable as it interacts with learning about leadership should be further examined. The canonical correlation results also revealed that the total leadership score, or the more leadership experiences the student athlete had coming into the course, the higher they rated the course in terms of facilitating leadership knowledge. This finding may suggest that the more leadership experience one has entering the course the more they may identify with the topics discussed and be interested in their application to the sport context.

It is also important to recognize what was not found in the canonical analyses. While the analysis was significant, the predictor set was weakly correlated to the course evaluation perceptions. In particular, there were no clear demographic characteristics (gender, time since taking the course, view of leadership ability) that distinguished between perceptions of the effectiveness of the course. This lack of finding is important as it demonstrates the course was found to be highly valuable with all types of scholastic athletes. As such, creators of youth life skill development resources (e.g., NFHS Online Captains Course) may want to focus course content on the most salient youth identity that impacts their leadership (e.g., student-athlete, dual career athlete) to maximize the potential positive impact (Lupo et al., 2017).

While the course was seen as useful overall, the student-athletes taking it also offered a number of suggestions for improving it. Most notable, were the recommendations around resolving conflict, whether that be with teammates or one’s coach. This makes sense as the course does not emphasize dealing with conflict to a great degree. Recent research has also highlighted how dealing with conflict is a difficult area for athletes in general (Wachsmuth et al., 2017) and young athletes in particular (Partridge and Knapp, 2016). Dealing with conflict is difficult for adults, much less scholastic youth athletes who have less conflict resolution experience and who may be highly motivated to be accepted by their peers. While additional content on conflict resolution could be added, this may be an area of leadership development best targeted in a coach follow-up to the course.

A more difficult recommendation to implement was to make the course more interactive by having more than just videos and written exercises. Developers might take lessons from the video game industry and include options such “plan your own adventure” type activities or other interactive exercises where one responds to something asked online and gets immediate customized feedback. Of course, this process is constrained by budgetary concerns, although the techniques available for online instruction have greatly expanded in the years the course was developed.

### Understanding the Coaches Role in the NFHS Course

The findings regarding the motivation for taking the course were interesting in that almost all (93%) the participants signed up for the course because they were urged to by a coach or athletic director. This emphasizes the importance the coach plays in foster youth leadership development. It also suggests that if one wants to expand the number of youth taking the course calls need to be made to coaches and administrators versus to students directly. Relative to this point, participants were asked if their coaches followed up, with 33% confirming a follow-up, but they were not asked to specify the type of follow-up provided. As such, this follow-up could have entailed anything from confirming that the athlete completed the course to an active conversation about course content. To maximize course effectiveness the course, developers recommended and included a brief guide for coaches to follow-up with the athlete after taking the course, as it was thought that doing so would allow the participants to contextualize the leadership knowledge conveyed to their particular context and coach approach. In future studies, it would be interesting to see what percentage of coaches use this resource and, if doing so, amplifies the effect of taking the course for the student-athlete leaders.

### Strengths and Limitations

This study had several strengths. First, while online education for both athletes and coaches are growing in popularity, seldom have evaluation studies been conducted to assess their effectiveness (Cotterill and Fransen, 2016). This study has done so and provided preliminary evidence for the effectiveness of the course. Second, a national sample of participants were randomly selected to participate with a wide variety of sports represented. This sample highlighted the potential utility of the course across sport, program, and geographical context. Third, due to the relative ease of access to the course and the decreased time demand on the coach, the positive evaluations seem to indicate the NFHS course as a viable option for time-strapped coaches who want to grow their athlete leaders. Additionally, while the course was designed for captains, the content can easily be used to develop informal leaders as well, allowing for all athletes to access this leadership development.

Relative to limitations, only self-reports of the course’s effectiveness were obtained and only from the student athlete participants. While the individual athlete’s perceptions and reflections on the course will undoubtedly play a role in their leadership behaviors, as is demonstrated in Table 6, Loughead (2017) highlights that at present, the field has yet to determine a “gold standard” for measuring athlete leadership behavior or quality. Additionally, Cotterill and Fransen (2016) highlight that in evaluating athlete leadership effectiveness both the coach and peers should be part of this assessment, compared to self-report alone. Thus, while it was important to understand the perceptions of the course effectiveness on leadership as “proof of concept,” further work will be needed in the future to track if and how the course affects leadership behaviors across a high school season. Related to this point, assessing the opinions of coaches and teammates in evaluating leadership behavior is important both pre- and post-course.

A second limitation was the reflective measure used. While we were able to establish that it was reliable, Kember et al.’s (2000) scale has not been used in the sport context. It needs to be validated to determine that it is a good measure of athlete reflection or if another measurement may be more appropriate. Finally, while the survey used in this study had good face validity, it was intended for one time use and extensive validation had not occurred. The amount of time from course completion to survey completion was also a weakness, with over half (56.3%) of the students evaluating the course 6 + months after completing it. Although we found no correlation between the amount of time from course completion to the overall course effectiveness rating, this time delay in response could introduce recall bias.

### Conclusion and Future Research Directions

The results of this study highlighted the potential value of the NFHS Online Captains Course to help scholastic athletes understand the necessary elements of athlete leadership within a sport context. The athletes overall positive evaluation of the course, as well as the ease with which they cited being able to translate elements of the course to their leadership on the playing field highlights this course may serve as critical “first exposure” to building leadership skills. For coaches with multiple competing time demands, but the desire to build athlete leaders, the NFHS course may be a meaningful resource to employ with their teams formal and informal leaders.

While the results of this investigation are encouraging, additional research is warranted. Most important is the need to conduct an intervention study where athlete leadership is assessed prior to and after taking the course with a group of course participants compared to non-course participants. It would be useful if future investigators longitudinally assessed athlete leadership development across a sports season, examining how taking the course interacted with the leadership experience to influence leadership behavior. Additionally, as previously stated, getting other stakeholder views (e.g., coaches, teammates) of the course participant’s leadership would be useful. Since athlete leadership is a shared practice (Duguay et al., 2019) and might be best viewed through the lens of a social identity approach to leadership (Worley et al., 2020), there might be a need to ensure that researchers examine not just the individual development that may occur from leadership courses, but the critical ways in which these courses can also serve as a starter to shared conversation and continued development in the networks of relationships that exist on a team.

There has been considerable research conducted in recent years focusing on informal peer leadership in sport (e.g., Fransen et al., 2014, 2015). It would be useful if future investigators assessed the influence of the course not only on student-athletes who are formal team captains, but those who occupy informal leadership roles on teams. For example, one might have entire teams of young athletes take the online course and then assess how the course experience influenced their experiences of leading and being led.

Finally, exploring the role of the coach in cultivating youth leadership is important. Why do some coaches recommend athletes take the course while others do not? What do coaches expect the course to accomplish for the young athletes who take it? And, how often, and in what ways, do coaches follow-up on what is taught in the online course? Further expansion on the decision to use the NFHS Online Leadership Course, and the way this course is specifically utilized with teams, should be explored to better understand how to maximize the impact of this course.

## Data Availability Statement

The raw data supporting the conclusions of this article will be made available by the authors, without undue reservation.

## Ethics Statement

The studies involving human participants were reviewed and approved by Human Research Protection Program, Michigan State University. Written informed consent to participate in this study was provided by the participants’ legal guardian/next of kin and the athletes themselves.

## Author Contributions

LW helped plan the study, conducted the data collection, analyzed the data, and wrote the majority of the manuscript submitted. DG helped plan the study, helped interpret data analysis, and wrote part of the manuscript submitted. Both authors contributed to the article and approved the submitted version.

## Conflict of Interest

The authors declare that the research was conducted in the absence of any commercial or financial relationships that could be construed as a potential conflict of interest.
